# Tailoring the Crystallinity of Ultrasonically Welded Interfaces in Glass Fiber-Reinforced Thermoplastic Composites

**DOI:** 10.1021/acsaenm.5c00281

**Published:** 2025-05-01

**Authors:** Md Asmat Ullah, Wencai Li, Miriam Siebenbuerger, Felipe Savella, Genevieve Palardy

**Affiliations:** † Department of Mechanical and Industrial Engineering, Louisiana State University, 3261 Patrick F. Taylor Hall, Baton Rouge, Louisiana 70803, United States; ‡ Department of Mechanical Engineering, University of Michigan, 2350 Hayward Street, Ann Arbor, Michigan 48109, United States; § Center for Advanced Microstructures and Devices, 5779 Louisiana State University, Baton Rouge, Louisiana 70803, United States

**Keywords:** thermoplastic composites, polypropylene, glass fiber, ultrasonic welding, crystallization

## Abstract

Ultrasonic welding (USW) is a fast and effective method for joining thermoplastic composites, offering excellent bonding strength that results in lightweight, durable structures, making it a cost-effective alternative to traditional joining techniques. The crystallinity at the weld interface impacts the mechanical properties and chemical resistance of the joint. The crystallization mechanisms at the bonded interface remain inadequately understood for the USW process, especially given its rapid cooling rates. This study investigates the use of polypropylene (PP) and multiwalled carbon nanotube (MWCNT)/PP films for ultrasonic welding of glass fiber (GF)/PP adherends, focusing on how process parameters influence the crystallinity degree, crystalline phases, crystallite size and spacing, lamellar structure and anisotropy, and molecular changes at the welded interface. Differential scanning calorimetry (DSC), scanning electron microscopy (SEM), Fourier transform infrared spectroscopy (FTIR), X-ray diffraction (XRD), and small-angle X-ray scattering (SAXS) were employed to gain a better understanding of crystalline structure at the interface. Four different sets of welding force and amplitude were tested: (1) 500 N, 38.1 μm, (2) 500 N, 54.0 μm, (3) 1500 N, 38.1 μm, and (4) 1500 N, 54.0 μm. The study demonstrated that despite fast cooling rates obtained during the process, higher welding force and amplitude significantly enhanced crystallinity, achieving 55% for welds with pure PP films and approximately 60% for MWCNT/PP films, compared to 35% and 41%, respectively, before welding. Notably, amplitude influenced the crystallinity at the welded interface more significantly compared to the force. SAXS experiments revealed that both pure PP and MWCNT/PP films exhibited isotropic structures prior to welding, but distinct anisotropy after welding. These findings suggest that strain-induced crystallization results from the welding process, with the degree of anisotropy correlating with the applied welding force and amplitude.

## Graphical abstract

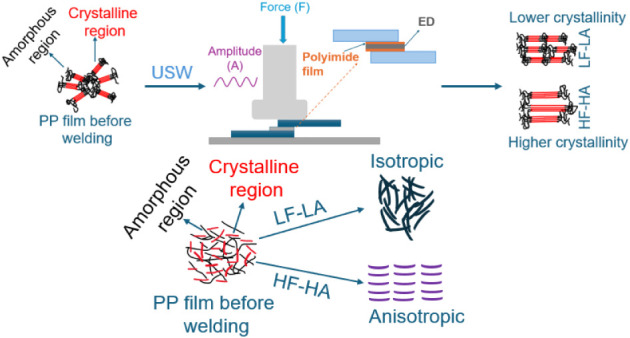

## 1 Introduction

The demand for lightweight yet durable structures has been increasing in several industries, such as automotive, aerospace, construction, naval, and energy. To meet this demand, cost-effective materials and manufacturing methods are continuously sought. Over recent decades, thermoplastic composites (TPCs) have emerged as compelling alternatives to traditional metals and thermoset composites due to their low weight, rapid processing, potential for recyclability, and corrosion resistance.^1–3^ Moreover, they enable fusion bonding, which presents distinct advantages over conventional joining methods like mechanical fastening and adhesive bonding. Notably, it avoids stress concentration introduced by drilled holes and reduces joining time without the need for extensive surface preparation and curing. Among fusion bonding techniques, ultrasonic welding (USW) stands out for its short process duration, minimal production costs and energy consumption, as well as its potential for automation.^4,5^ This process uses high-frequency vibrations to fuse materials via surface friction and localized viscoelastic heating at the contact interface.

To direct heat generation at the interface, it is common to use energy directors (EDs), such as thermoplastic films or protrusions, to locally promote friction and temperature rise.^4,6–8^ In some cases, such as spot-welding, it was shown the EDs were not necessary to produce high-quality joints,^4,9,10^ However, ED films were also demonstrated to provide multifunctionality as nanocomposite heating elements for conduction and resistance welding, and for damage monitoring of ultrasonically welded joints.^11–15^ Notably, in previous work, the authors demonstrated multifunctionality of multiwalled carbon nanotubes (MWCNTs)-based nanocomposite films to facilitate heat generation during welding and real-time structural health monitoring via electrical resistance fluctuations,^13,14,16^

Crystallinity is one of the key determinants for many semicrystalline thermoplastic properties, like chemical resistance, thermal and electrical conductivity, stiffness, shear strength, interfacial strength, and fracture toughness.^17–20^ Typically, crystalline thermoplastics possess higher chemical resistance and solvent resistance than amorphous polymers due to their semicrystalline structure.^21,22^ Talbott et al. explored the mechanical characteristics of poly ether ether ketone (PEEK), showing that reduced crystallinity correlates with decreased elastic modulus, tensile strength, and shear strength.^23^ Similarly, prior studies have noted the advantageous effect of crystallinity on the tensile properties of polyphenylene sulfide (PPS) and its composites.^19,24,25^ Grouve et al. found that crystallinity positively influenced the interfacial shear strength of carbon fiber-reinforced PPS and PEEK.^20^ On the other hand, fracture toughness (Mode I) was reported to increase with a decrease of crystallinity.^26–28^ Previous research efforts on USW of TPCs have primarily focused on understanding heating mechanisms and process parameters influencing weld strength.^9,29–33^ Key parameters such as vibration amplitude, hold time, and welding force were recognized as important factors affecting weld performance, with higher forces leading to shorter welding times and lower welding energies.

There is limited research in the literature investigating the effect of welding parameters on the crystallinity and crystalline structure at TPC joints interface, especially for USW. Koutras et al. characterized the crystallinity of CF/PPS ultrasonically welded joints using differential scanning calorimetry (DSC) and wide-angle X-ray diffraction (WAXD).^19,34^ The effect of welding force and amplitude, and their corresponding cooling rate, were considered. It was observed that high force and amplitude values led to the lowest crystallinity (<2.5%, amorphous PPS), with a cooling rate between 41 °C/s and 19 °C/s as time increased. For low force and amplitude, crystallinity ranged from 14% to 18%, with slower cooling rates between 17 °C/s and 4 °C/s. It was suggested that the PPS welds could crystallize at low welding force and amplitude despite the high cooling rates measured at the interface, due to strain-induced crystallization (SIC) under ultrasonic vibrations. This was not the case for welds produced at high force and amplitude because the melted PPS at the interface likely did not spend enough time under high strain rates for SIC to occur. Overall, it is expected that the cooling rate will have an impact on the crystallinity, but the threshold rate leading to amorphous structure generally depends on the polymer.^35,36^

The addition of nanoparticles can affect polymers by influencing thermal and electrical conductivities, and the growth and arrangement of crystalline structures.^17,37–40^ The nanoparticles can serve as heterogeneous nucleating agents, causing the formation of smaller and more uniformly distributed crystallites. This results in an increase in the overall crystallinity of the material. Various researchers have extensively studied the impact of carbon nanotubes (CNTs) on the crystallization behavior of polymer nanocomposites. The effect of CNT loading on the crystallinity of polypropylene (PP) varies in the literature, with some studies reporting no change, slight decline, or a small increase. For instance, previous experiments demonstrated that the addition of MWCNTs increased crystallinity by up to 6% with 1 wt % MWCNT.^37^ It was found that PP crystallinity increased by up to 5% with the addition of 1 wt % CNT, but decreased with the addition of 5 wt % CNT.^38^ They assumed this result was the consequence of blocking the PP chains, which occurred from the high percentage of CNTs. In summary, CNT weight fraction affects crystallinity of CNT/PP composites, but it is unclear how the ultrasonic welding process could impact the crystallization behavior of polymer nanocomposites. Nanocomposites demonstrated potential as multifunctional films for ultrasonically welded TPC joints, enabling strain sensing, structural health monitoring, and heating elements for resistance welding or disassembly.^13–15^ Therefore, it is important to assess the extent of changes in crystallization behavior at the interface between pure and nanocomposite thermoplastic films.

Understanding the polymer state at the weld interface is necessary due to its effect on final properties. Thus, this study aims to analyze the effect of welding parameters on several fundamental characteristics at the interface of ultrasonically welded glass fiber (GF)/PP joints with both pure PP films and MWCNT/PP films: degree of crystallinity, crystalline phases, crystallite size and spacing, lamellar structure and anisotropy, and molecular changes. In this paper, characterization results from scanning electron microscopy (SEM), DSC, WAXD, Fourier-transform infrared (FTIR) spectroscopy, and small-angle X-ray scattering (SAXS) will first be presented. Then, outcomes will be discussed to assess the influence of welding parameters (force and amplitude) on crystallization characteristics at the interface, strain-induced crystallization, and USW process parameter selection to tailor interfacial crystallinity.

## 2 Materials and Methods

### 2.1 Materials

In this study, GF/PP laminates were manufactured with eight unidirectional (UD) GF/PP prepreg layers (IE 6030, 60% fiber volume fraction, and PP copolymer), provided by Avient (formerly PolyOne, Avon Lake, OH, USA). For the ultrasonic welding process, a PP or MWCNT/PP film was placed at the interface between the parts to be joined to act as an energy director or provide multifunctionality. PP pellets, provided by Goodfellow (Coraopolis, PA, USA), were used to create pure PP films. PP pellets containing 20 wt % MWCNT were purchased from Cheap Tubes Inc. (Grafton, VT, USA) as a masterbatch for the preparation of nanocomposite films. The MWCNTs possessed an outer diameter between 10 and 20 nm, and a length of 10 to 30 μm.

### 2.2 Film and Laminate Manufacturing

The GF/PP laminates were manufactured by stacking prepreg layers in a [0]_8_ sequence, then applying consolidation in a 75-ton heated press (Dake, Grand Haven, MI, USA) at 180 °C and 1 MPa pressure for 15 min, followed by cooling to room temperature. The final laminate thickness was approximately 1.8 mm, with a void content below 1.5%. The process of preparing 20 wt % MWCNT/PP films involved compression molding. The MWCNT pellets were subjected to compression molding in a laboratory heated press (Dake, Grand Haven, MI, USA), at 0.8 MPa and 180 °C for 15 min and followed by cooling to room temperature to get films approximately 0.5 mm thickness.

### 2.3 Ultrasonic Welding Procedure

Specimens were joined in a single-lap configuration (Figure 1) using a displacement-controlled Dynamic 3000 ultrasonic welder from Rinco Ultrasonics (Danbury, CT, USA). The welder operated at a frequency of 20 kHz and a maximum power of 3000 W, with a booster gain of 1:1.5. To create the GF/PP joints, 0 wt % MWCNT/PP (pure PP) films or 20 wt % MWCNT/PP films were placed between the GF/PP samples to act as “energy directors” (EDs) at the interface. The welding process consists of a vibration phase, following by a solidification phase. During the vibration phase, a 1000 N force and a 38.1 μm vibration amplitude were applied. For the solidification phase, a 1000 N force was applied for 4000 ms. The welding process was controlled by adjusting the vertical displacement of the circular sonotrode (40 mm diameter), set at 60% of the initial film thickness during the vibration phase for all experiments, based on previous studies with GF/PP.^13^ Those welds were first used for general microstructural and crystallinity characterization at the welded interface.

**Figure 1:**
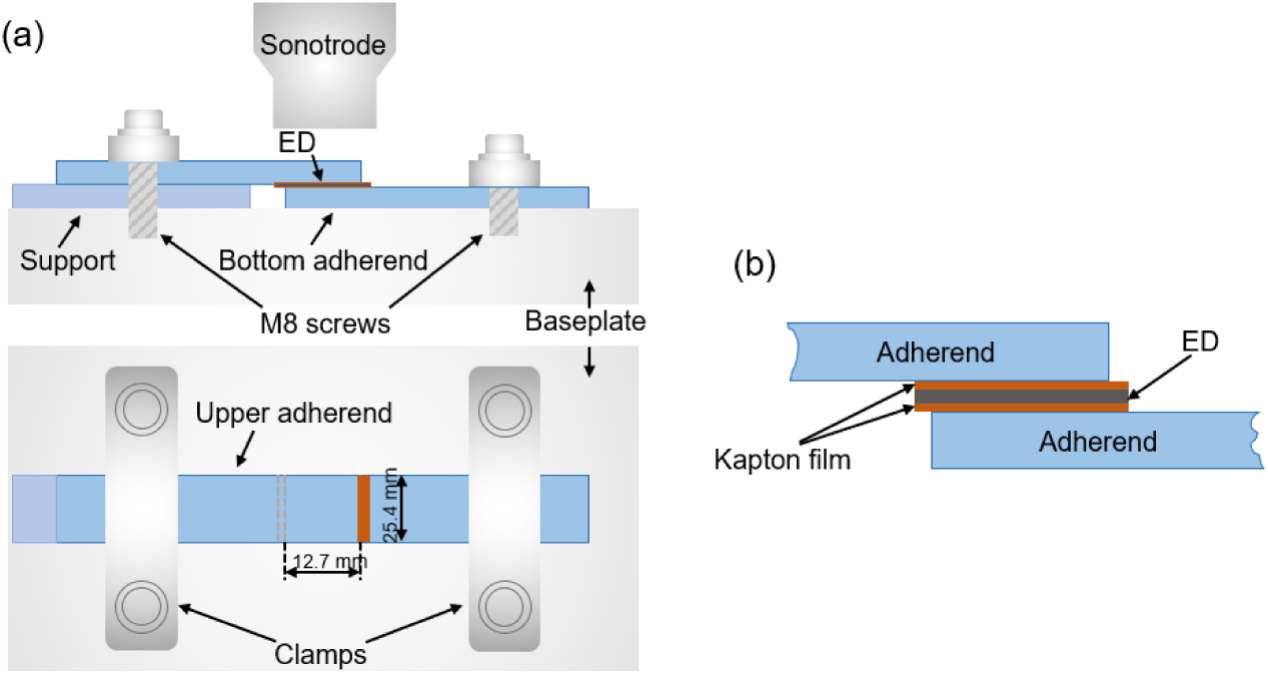
(a) Welding setup utilized for conducting experiments with single lap shear samples. The components in the schematic are labeled as follows: 1. Sonotrode, 2. Lower sample clamp, 3. Upper sample clamp, and 4. Sliding platform. (b) Schematic of the specimen setup designed for removing the energy director from the welded interface, allowing direct crystallinity measurements. Note that the dimensions in the schematic are not depicted to scale.

Then, to test the effect of ultrasonic welding process parameters on the crystallization behavior at the interface, four different sets of welding force and vibration amplitude were used, as summarized in Table 1. In this experiment, 0 wt % MWCNT/PP (pure PP) or 20 wt % MWCNT/PP ED films were used. To isolate the interface, the MWCNT/PP films were placed between two polyimide films (Kapton, purchased from American Durafilm, Holliston, MA, USA), each with a thickness of 25 μm (Figure 1b). These polyimide films served as a barrier to prevent direct contact between the GF/PP samples and the MWCNT/PP films. After the USW process, the MWCNT/PP films were carefully separated from the polyimide films for further characterization using DSC, WAXD, FTIR, or SAXS. It is to be noted that the polyimide films placed at the interface may affect heat conduction between the ED and adherends during the welding process, potentially altering cooling profile and crystallization rate. While the films may slightly shift thermal gradients, we expect the overall crystallinity trends to remain similar, allowing comparison between different welding parameters.

**Table 1:** Ultrasonic Welding Process Parameters to Study Crystallization Behavior at the Interface

Specimen Name	Welding Force (N)	Vibration Amplitude (μm)	Travel (mm)	Solidification force/duration (N/ms)
Low Force/Low Amplitude (LF-LA)	500	38.1	0.3	1000/4000
Low Force/High Amplitude (LF-HA)	500	54	0.3	1000/4000
High Force/Low Amplitude (HF-LA)	1500	38.1	0.3	1000/4000
High Force/High Amplitude (HF-HA)	1500	54	0.3	1000/4000

Temperature measurements were acquired during the USW process with a type K thermocouple based on an approach described in a previous study.^41^ The temperature curves showed that the cooling rate decreased over time. The slope was first computed at all time steps using a backward differentiation method. It is presented in Figure S1 for LF-LA and HF-HA cases during the cooling phase below the melting temperature (165 °C). Then, to provide an overall approximation of cooling rates, three temperature ranges below the melting temperature showing a quasi-linear behavior were selected: (1) 165 °C (melting temperature) to 90 °C, (2) 90 to 60 °C, and (3) 60 to 40 °C.^19^ For LF-LA, the cooling rates were thus estimated as 39.5 °C/s, 10.8 °C/s, and 2.1 °C/s, respectively. For HF-HA, the cooling rates were 47.9 °C/s, 13.7 °C/s, and 0.8 °C/s.

### 2.4 Scanning Electron Microscopy (SEM)

In this study, SEM analysis was employed to investigate the general morphological characteristics of welded joints formed with pure PP and MWCNT/PP films. Scanning electron imaging was conducted using a ThermoScientific Helios G5 CXe Plasma focused ion beam (FIB) SEM. Prior to observation, the specimens were coated with a thin layer of gold using an EMS550X sputter coater at 25 mA and a vacuum of 1 × 10^–1^ mbar for 2 min. The SEM utilized an accelerating voltage of 15 kV for imaging. Samples were initially embedded in epoxy resin molds. They were then subjected to a grinding process using SiC abrasive papers with grit sizes of 180, 360, 600, 800, and 1200. Following the grinding step, the samples underwent polishing using diamond solutions of 6 and 1 μm on polishing pads.

### 2.5 Differential Scanning Calorimetry (DSC)

A PerkinElmer Differential Scanning Calorimeter 4000 was used to determine melting enthalpy and degree of crystallinity of the different samples. Crystallinity was measured for three types of samples: (1) pure PP and MWCNT/PP films before welding, (2) films collected from the interface after welding, and (3) welded interfaces with film. For the latter, the welded joint was cut, then the interface was collected with a sharp blade for DSC experiments. All samples weighed between 5 mg and 10 mg, sealed in a 5 mm diameter aluminum pan. The pan was placed in the DSC with a second empty aluminum pan for reference. The samples were heated up to 200 °C at 10 °C/min, then remained at 200 °C for 2 min. They were cooled down to room temperature at 10 °C/min. After 2 min at room temperature, the cycle was repeated a second time. The crystallinity, *X*_c_ (%), was calculated from the first DSC cycle using eq 1:

1 $$X_{c}=\frac{\Delta H_{m}}{\Delta H_{m}^{o}\times \left(1-\alpha \right)}\times 100$$

where Δ*H*_m_ is the measured specific melting enthalpy (J/g), $\Delta H_{m}^{o}$ is the specific melting enthalpy of an ideal crystal (209 J/g), and α is the MWCNT weight fraction. Representative DSC curves for all cases before and after welding are shown in Figures S2–S5.

### 2.6 Fourier-Transform Infrared (FTIR) Spectroscopy

FTIR analyses were performed to assess molecular changes using a Nicolet 6700 FTIR spectrometer (Thermo Fisher Scientific, USA) at a constant temperature of 23 °C. Spectra were obtained in attenuated total reflection (ATR) mode within the range of 400 to 4000 cm^–1^ to assess the crystallinity of polypropylene. In this experiment, only pure PP films before welding and PP films collected from the interface after welding under different USW parameters were characterized, as no spectra could be obtained from the MWCNT/PP films. Each spectrum was an average of 32 scans, with a resolution set at 0.48 cm^–1^. The reported data represents the average and standard deviation of three measurements taken from three distinct locations on a single sample. ATR-FTIR measurements employed both diamond and germanium single bounce crystals, with a reflection angle (θ) of 42°. The diamond crystal, possessing a refractive index (nDia) of 2.4, was utilized alongside polypropylene (nPP), which has a refractive index of 1.49.

### 2.7 Wide-Angle X-Ray Diffraction (WAXD)

A WAXD analysis was conducted to investigate the crystal phases present within the interfaces, crystallize size and spacing, and the degree of crystallinity. WAXD measurements were analyzed for two types of samples: (1) pure PP and MWCNT/PP films before welding, and (2) films collected from the interface after welding under different USW parameters (as shown in Figure 1b). WAXD experiments were carried out with a PANalytical Empyrean multipurpose diffractometer, operating at 45 kV and 40 mA. Cu Kα radiation with a wavelength of *k* = 1.5418 Å was employed, with a stepwise scan ranging from 5° to 60° in the scattering angle (2θ). The acquired data was processed with PANalytical X′Pert software.

### 2.8 Small-Angle X-Ray Scattering (SAXS)

SAXS measurements were performed to investigate lamellar structure and anisotropy for two types of samples: (1) pure PP and MWCNT/PP films before welding, and (2) films collected from the interface after welding under different USW parameters (as shown in Figure 1b). SAXS was measured with the beamline at the Center for Advanced Microstructures and Devices (CAMD) synchrotron with a Xenocs Ganesha instrument. In this experiment, a Cu Kα laboratory X-ray source (Genix from Xenocs) with a wavelength of 1.54 Å was used. Namely, the scattering measurements were performed at room temperature over a range of *q* from 0.004 to 1.2 Å^–1^ (*q* = (4π/λ)­sin θ where λ and 2θ are the wavelength of the X-ray source and the scattering angle, respectively). SAXS experiments were conducted using a setup where the X-ray beam was directed perpendicular to the sample interface, ensuring that the scattering data exclusively captured interfacial structures. The sample-to-detector distance was adjusted accordingly to achieve optimal resolution. Since the measurements focused solely on the interfaces, no distinct bulk material directionality was considered. The 2D data reduction and radial averaging over 5-degree steps were performed with the Igor Pro Nika package of Jan Ilavsky (APS).

The structural characterization of semicrystalline materials relies on both classical and recent methodologies, particularly utilizing electron correlation functions. In this study, structural parameters were derived using previous techniques.^42,43^ The one-dimensional electron correlation function, γ­(*r*), was computed from two-dimensional scattering patterns, facilitating analysis in reciprocal space. Experimental curves underwent preprocessing steps including smoothing with moving average filtering and extrapolation to low and high *q* values. At higher angles, a function incorporating positive and negative deviations from Porod’s law was fitted to experimental data for the scattering intensity as a function of scattering vector (*I*(*q*)) (eq 2), allowing determination of parameters such as the thickness of transition layers:

2 $$\underset{q\to \infty }{\lim}I\left(q\right)=I_{\mathrm{fl}}+\frac{A_{\mathrm{p,q}}}{q^{4}}\exp\left(-\sigma_{\ln}^{2}q^{2}\right)$$

where *I*_fl_ is a constant background scattering due to electron density fluctuations within the phases, *A*_*p,q*_ is the Porod constant, and *σ*_ln_ is a parameter characterizing the thickness of the transition layer.

Morphological parameters of lamellar stacks were determined from the linear correlation function γ­(*r*), which was obtained through cosine transformation of Lorentz-corrected SAXS intensity distributions. This analysis provided insights into parameters such as long period (*L*_p_) and average interface thickness (*D*_0_). Additionally, the linear crystallinity (*X*_l_) was deduced from γ­(*r*), offering information about the relative fractions of crystalline and amorphous phases within the lamellar stacks with eq 3:

3 $$X_{l}\left(1-X_{l}\right)=\frac{D_{0}}{L_{p}}$$

The dimensions of the crystalline (*l*_c_) and amorphous (*l*_a_) layers within the stacks were derived through the subsequent analysis with eqs 4 and 5:

4 $$l_{c}=X_{l}L_{p}$$

5 $$l_{a}=\left(1-X_{l}\right)L_{p}$$

## 3 Results and Discussion

### 3.1 Interface Morphology and Crystallinity Measurements via DSC

The SEM images presented in Figure 2 illustrate the evolution of the interface morphology from pure PP film to high wt % MWCNT (25 wt %). Notably, the interface between the materials was not clearly discernible in the absence of MWCNT (0 wt %), whereas with increasing MWCNT content, the interface became more distinct. This observation suggests a pronounced demarcation between the MWCNT films and the GF/PP adherends, indicating limited diffusion of the PP chains into the GF/PP adherends during the USW process, likely resulting from an increased viscosity due to the presence of MWCNTs.^13,17^

**Figure 2:**
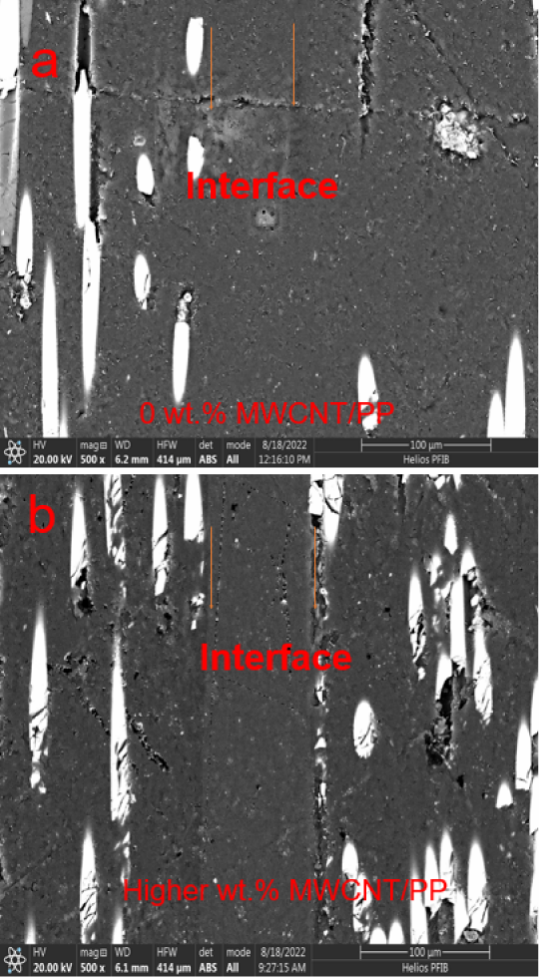
Representative SEM images for GF/PP joints welded with (a) pure PP films, (b) high wt % MWCNT/PP films. All scale bars: 100 μm.

To investigate the impact of ultrasonic welding process parameters on the degree of crystallinity (*X*_c_) at the welded interface for GF/PP joints, four distinct sets of parameters involving welding force and vibration amplitude were employed (see Table 1). The crystallinity values corresponding to each parameter set are summarized in Figure 3. Employing a welding force of 500 N and a vibration amplitude of 38.1 μm (LF-LA) resulted in lower crystallinity at the interface compared to conditions with a welding force of 1500 N and a vibration amplitude of 54 μm (HF-HA). For example, under the former conditions, pure PP films displayed a crystallinity of 34.5 ± 2.9%, which increased to 55.0 ± 3.6% under HF-HA conditions. Similarly, in the case of MWCNT/PP films, the crystallinity rose from 41.3 ± 2.5% to 59.3 ± 2.5% when transitioning from lower to higher welding forces and vibration amplitudes.

**Figure 3:**
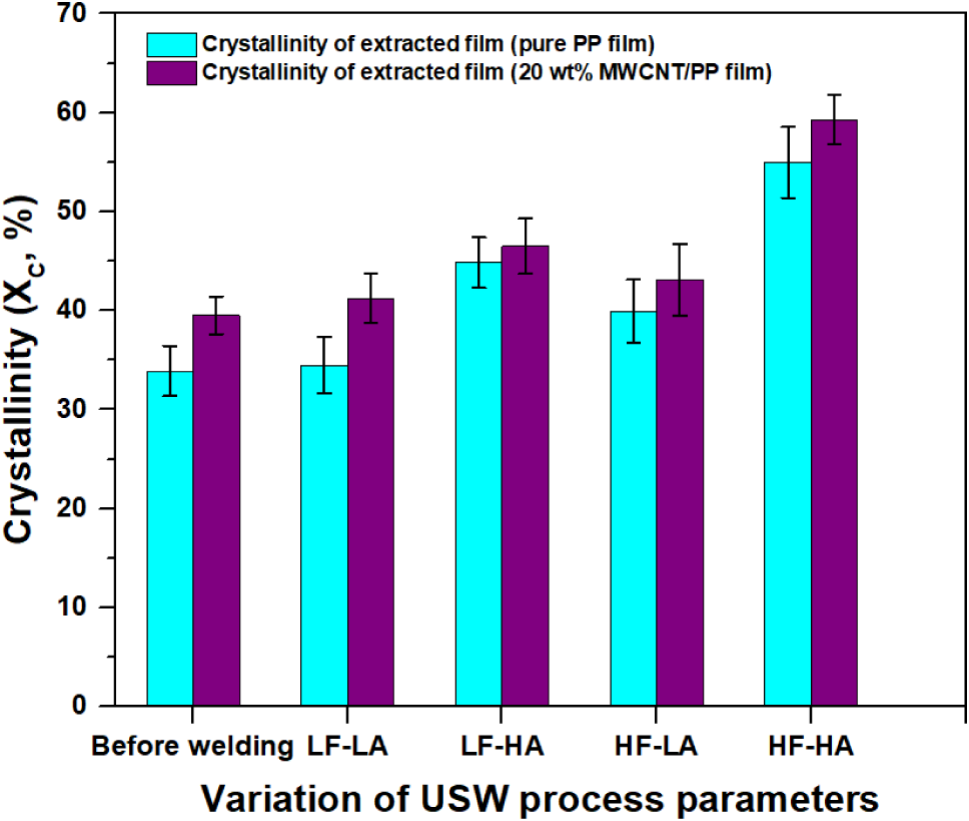
Crystallinity of PP films and 20 wt % MWCNT/PP films collected from the interface after welding at various welding parameters determined from standard DSC measurements. Here, LF-LA (500 N, 38.1 μm), LF-HA (500 N, 54 μm), HF-LA (1500 N, 38.1 μm), and HF-HA (1500 N, 54 μm).

### 3.2 Molecular Changes and Crystallinity via FTIR Spectroscopy

A method for assessing crystallinity using FTIR spectroscopy was previously described in the literature by Burfield and Loi,^44^ Huy et al.,^45^ and Kilic et al.^46^ In this technique, the degree of crystallinity (*X*_c_) is determined by analyzing the ratio of peak heights at specific wavenumbers. Specifically, the peak at 998 cm^–1^ (Figure 4a) is indicative of the crystalline phase, with its height increasing proportionally with the degree of crystallinity. Since both the α and β crystal phases of polypropylene exhibit similar FTIR spectra, the peak height at 998 cm^–1^ serves as a measure of overall crystallinity but does not distinguish between these phases. The 974 cm^–1^ peak originates from the amorphous phase, meaning it is inherently correlated with the overall crystalline-to-amorphous ratio. To more accurately assess crystallinity, the ratio of the peak heights at 998 cm^–1^ and 1375 cm^–1^ (associated with symmetrical C–H stretching of CH_3_ groups) is a more reliable measure, as suggested in the literature.^47^ To determine peak heights, a linear baseline correction is applied using measured points at adjacent wavenumbers. The vertical drop is then measured from the highest absorbance value within the specified range for each peak, ensuring a consistent and reliable method for crystallinity quantification.

**Figure 4:**
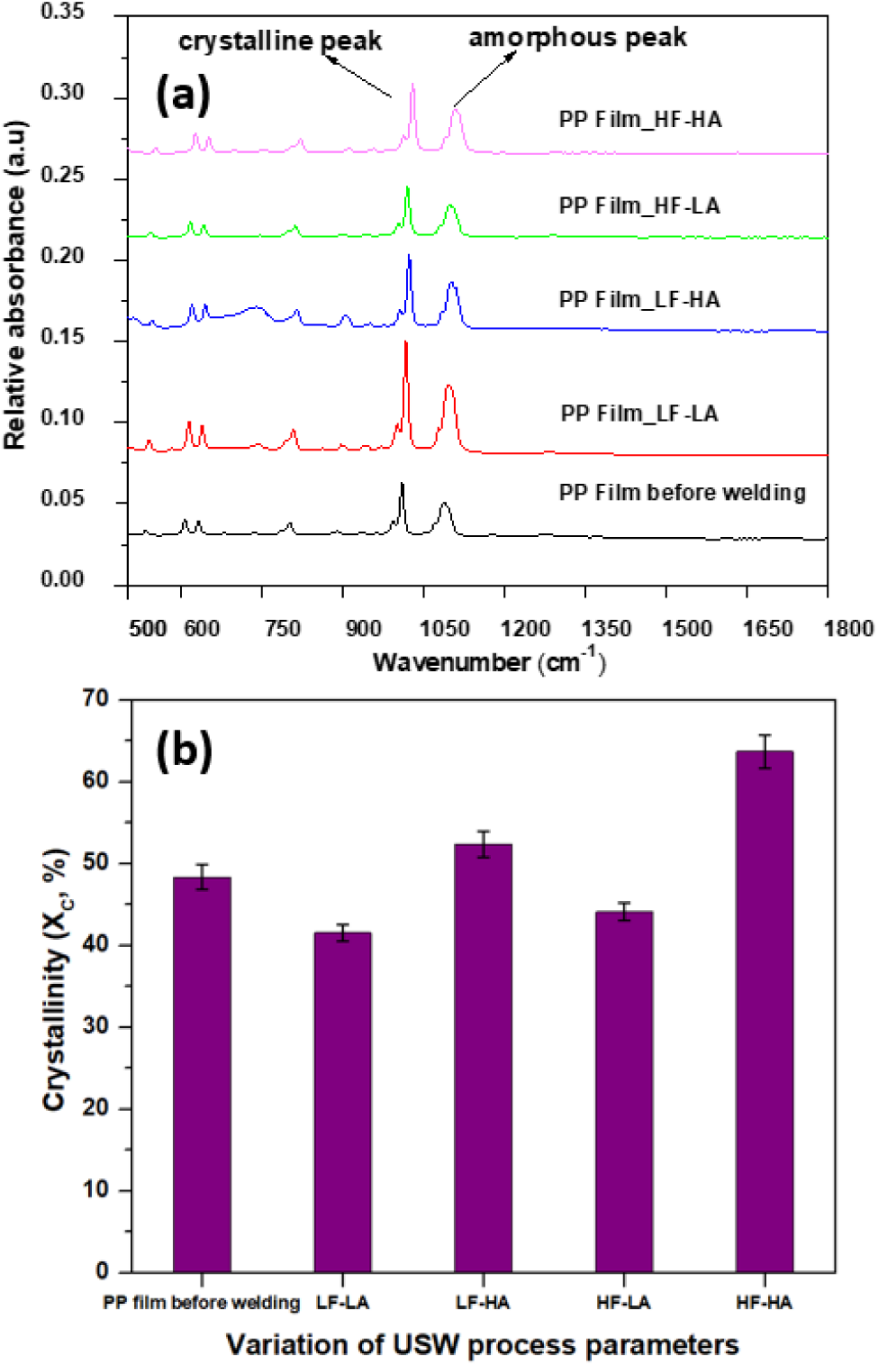
(a) FTIR Overlay spectra for PP films (before welding and collected from the interface after welding) and (b) crystallinity of PP films (before welding and collected from the interface after welding) determined from standard FTIR measurements. LF-LA (500 N, 38.1 μm), LF-HA (500 N, 54 μm), HF-LA (1500 N, 38.1 μm), and HF-HA (1500 N, 54 μm).

Similarly to the DSC analysis, FTIR analysis (Figure 4b) showed that when employing a welding force of 500 N and a vibration amplitude of 38.1 μm (LF-LA), lower crystallinity was obtained compared to conditions featuring a welding force of 1500 N and a vibration amplitude of 54 μm (HF-HA). For instance, pure PP films exhibited a crystallinity of 38.2% under the former conditions, whereas it increased to 60.5% under the latter conditions. It should be noted that crystallinity could not be accurately measured for MWCNT/PP films due to insufficient transmission.

### 3.3 Crystalline Structure and Crystallinity via WAXD

Figure 5 shows the wide-angle X-ray diffractograms of pure PP films before and after welding under various welding parameters. Both pure PP films before and after welding display characteristic diffracting peaks at 2θ = 13.9°, 16.7°, 18.3°, 21.6° and 42°, corresponding to the planes (1 1 0), (0 4 0), (1 3 0), (1 1 1), and (0 4 1) of its α-phase crystallite and exhibiting complete absence of the β-crystal form. Similarly, the integrated X-ray diffraction intensity of PP and MWCNT/PP nanocomposites demonstrates the dominance of the α-phase in both materials, suggesting that the addition of 20 wt % MWCNTs does not alter the crystalline structure of the PP matrix.^48^ Although the number of peaks and their positions remain consistent between PP and MWCNT/PP nanocomposite films, differences in the relative intensities of the peaks are observed, indicating potential variations in crystallinity or molecular arrangement.

**Figure 5:**
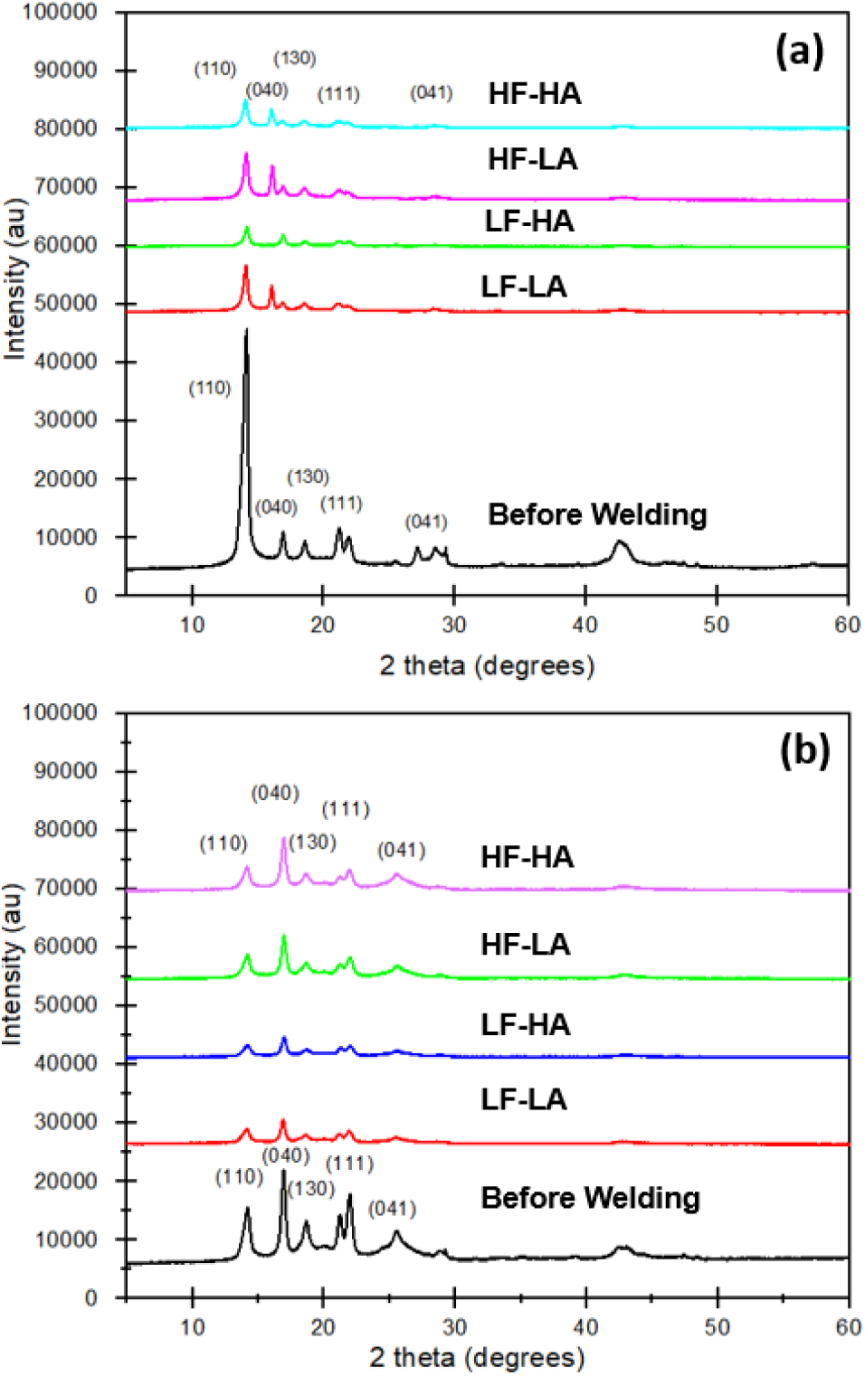
(a) WAXD curves of pure PP films before welding and after welding and (b) WAXD curves of 20 wt % MWCNT/PP films before welding and after welding. LF-LA (500 N, 38.1 μm), LF-HA (500 N, 54 μm), HF-LA (1500 N, 38.1 μm), and HF-HA (1500 N, 54 μm).

Figure 6 summarizes the crystallinity of the specimens welded under different parameters. Prior to welding, pure PP films displayed a crystallinity of 48.2%, while MWCNT/PP films exhibited a higher crystallinity of 54.4%. When a welding force of 500 N and a vibration amplitude of 38.1 μm (LF-LA) were employed, lower crystallinity was observed at the interface compared to conditions with a welding force of 1500 N and a vibration amplitude of 54 μm (HF-HA). Specifically, under the former conditions, pure PP films exhibited a crystallinity of 45.7%, whereas this value increased to 70.1% under the latter conditions. Similarly, MWCNT/PP films demonstrated a comparable trend, with crystallinity measuring 37.2% under low force and low amplitude (LF-LA) conditions and increasing to 68.1% under high force and high amplitude conditions (HF-HA). Overall, WAXD analysis results show a similar trend to DSC and FTIR for the crystallinity at different process parameters.

**Figure 6:**
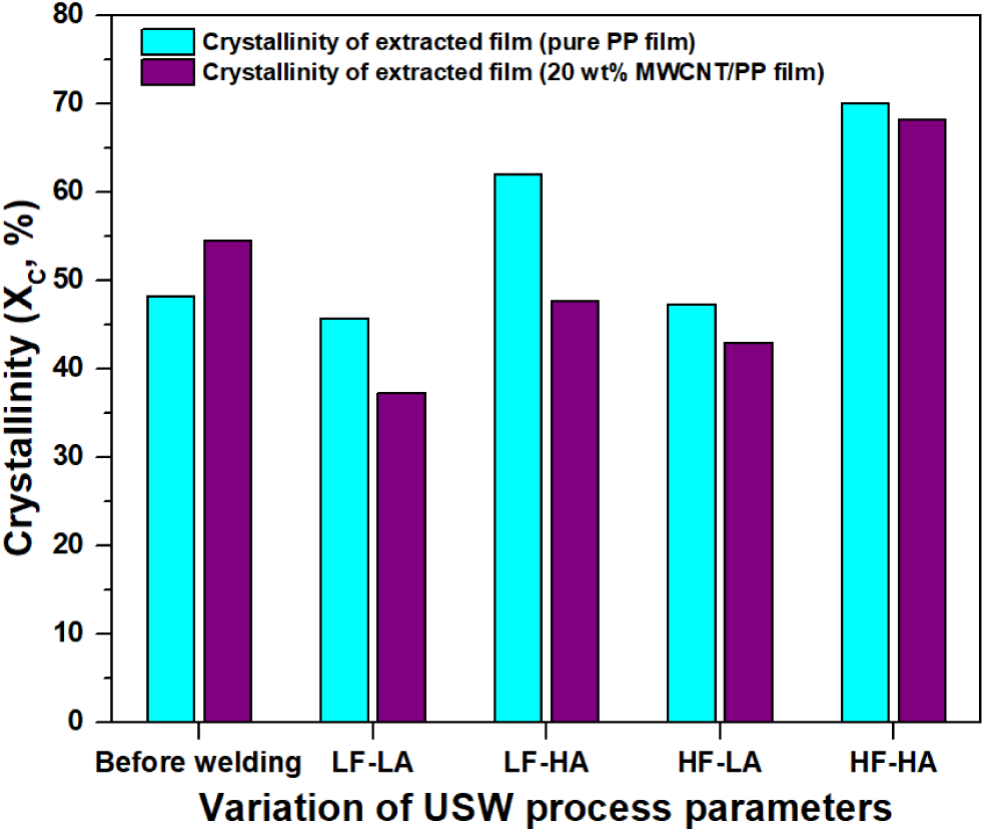
Crystallinity of PP films and 20 wt % MWCNT/PP films (before welding and collected from the interface after welding at various welding parameters) determined from standard WAXD measurements. LF-LA (500 N, 38.1 μm), LF-HA (500 N, 54 μm), HF-LA (1500 N, 38.1 μm), and HF-HA (1500 N, 54 μm).

WAXD analysis can provide further information about apparent crystallite size using the Scherrer eq 6:^49^

6 l(hkl)=kλ/β⁡cos⁡θ

where the correction factor (*k*) accounts for lattice distortion, *l­(hkl)* represents the crystal size perpendicular to the plane (*h k l*), λ denotes the applied wavelength, θ stands for the Bragg angle, and β represents the full-width-at-half-maximum (fwhm) scattering intensity. The relationship between the interplanar spacing (*d*) and the Bragg angle (θ) is described by eq 7, where *n* is assumed to be one and the other parameters are the same as those in eq 6:

7 d=nλ/2⁡sin⁡θ

Tables 2 and 3 provide a summary of the results from the WAXD analysis, detailing the crystal size normal to various planes. It was observed that lower welding force and amplitude (LF-LA) lead to a reduced crystal size along all planes (110, 040, 130, 111, 041) in both pure PP films and MWCNT/PP films, while higher force and amplitude (HF-HA) result in larger crystal sizes. Overall, no significant alteration in *d*-spacing values was detected due to variations in welding parameters.

**Table 2:** Crystal Size and *d*-Spacing of PP Films (Before Welding and Collected from the Interface after Welding) Determined from Standard WAXD Measurements in Different Plane Directions

Specimen Name	PP film before welding (nm)	PP-(LF-LA) (nm)	PP-(LF-HA) (nm)	PP-(HF-LA) (nm)	PP-(HF-HA) (nm)
Crystallite size normal to (110) plane	15.07	12.16	17.72	16.67	16.40
*d*-spacing (110)	0.63	0.58	0.62	0.60	0.62
Crystallite size normal to (040) plane	24.62	16.50	29.63	21.10	27.23
*d*-spacing (040)	0.52	0.47	0.55	0.52	0.54
Crystallite size normal to (130) plane	21.13	9.5	12.99	15.45	15.58
*d*-spacing (130)	0.48	0.41	0.50	0.47	0.47
Crystallite size normal to (111) plane	6.81	5.35	7.51	8.36	7.87
*d*-spacing (111)	0.42	0.36	0.41	0.40	0.42
Crystallite size normal to (041) plane	8.44	6.50	5.42	5.34	6.12
*d*-spacing (041)	0.21	0.21	0.21	0.21	0.21

**Table 3:** Crystal Size and d-Spacing of 20 wt % MWCNT/PP Films (Before Welding and Collected from the Interface after Welding) Determined from Standard WAXD Measurements in Different Plane Directions

Specimen Name	20 wt % MWCNT/PP film before welding (nm)	20 wt % MWCNT/PP (LF-LA) (nm)	20 wt % MWCNT/PP (LF-HA) (nm)	20 wt % MWCNT/PP (HF-LA) (nm)	20 wt % MWCNT/PP (HF-HA) (nm)
Crystallite size normal to (110) plane	12.80	9.18	10.20	13.68	13.18
*d*-spacing (110)	0.63	0.55	0.61	0.62	0.62
Crystallite size normal to (040) plane	19.68	14.08	20.88	18.29	18.02
*d*-spacing (040)	0.52	0.50	0.52	0.52	0.52
Crystallite size normal to (130) plane	13.01	12.56	7.52	4.23	7.40
*d*-spacing (130)	0.47	0.46	0.48	0.47	0.47
Crystallite size normal to (111) plane	7.90	6.50	7.12	6.42	5.98
*d*-spacing (111)	0.40	0.34	0.41	0.34	0.40
Crystallite size normal to (041) plane	15.74	8.50	9.30	3.50	6.94
*d*-spacing (041)	0.21	0.21	0.21	0.21	0.21

### 3.4 Lamellar Structure and Anisotropy via SAXS

Figures 7 and 8 display representative 2D SAXS and WAXS images of PP and MWCNT/PP samples, respectively, obtained under typical experimental conditions. The analysis of the scattering images followed a standard procedure: the 2D data were azimuthally averaged from the beam center, normalized to the incident X-ray beam intensity monitored by an ionization chamber placed before the sample, and further corrected for background contributions during empty sample runs.^50,51^

**Figure 7:**
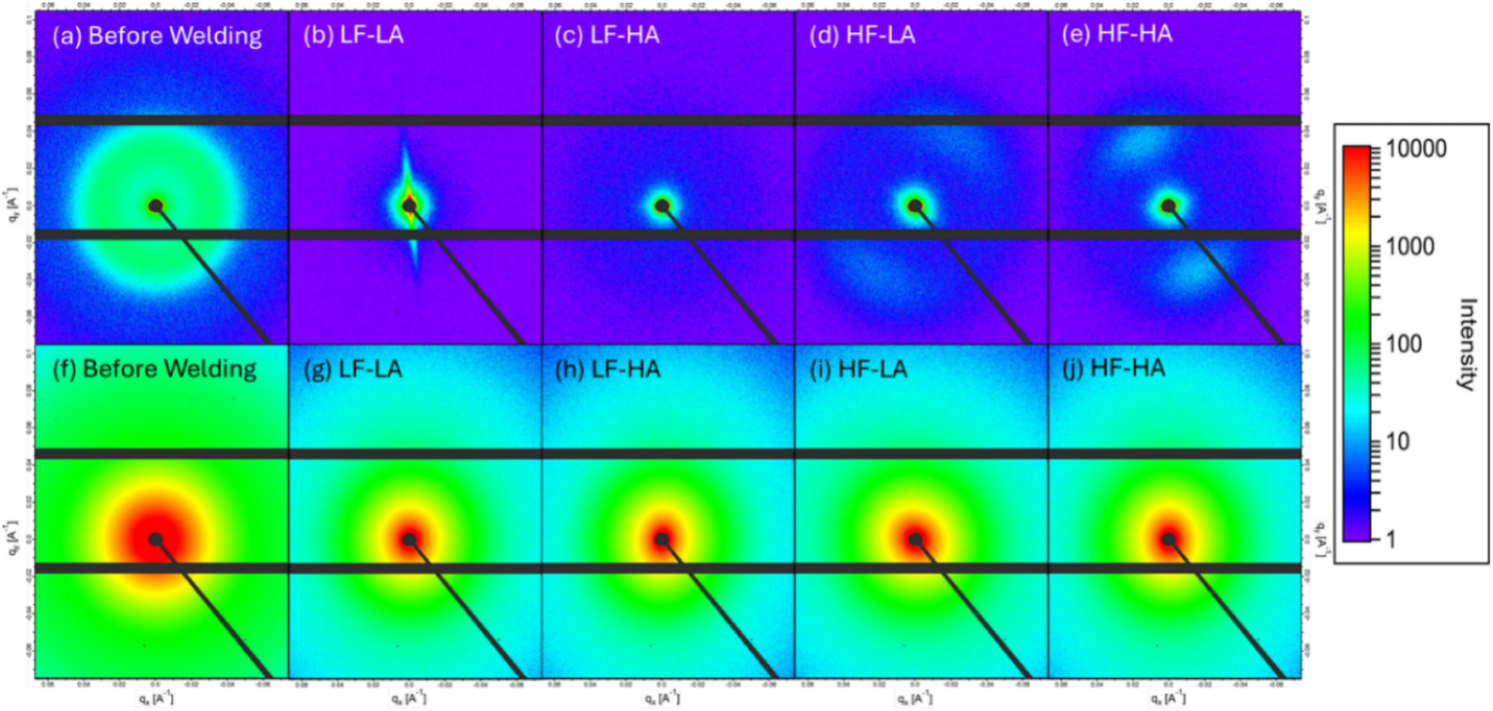
2D SAXS images of PP films (before welding and collected from the interface after welding) in the top row (a–e) and 2D images of MWCNT/PP (f–j) films (before welding and collected from the interface after welding) in the bottom row. LF-LA (500 N, 38.1 μm), LF-HA (500 N, 54 μm), HF-LA (1500 N, 38.1 μm), and HF-HA (1500 N, 54 μm).

**Figure 8:**
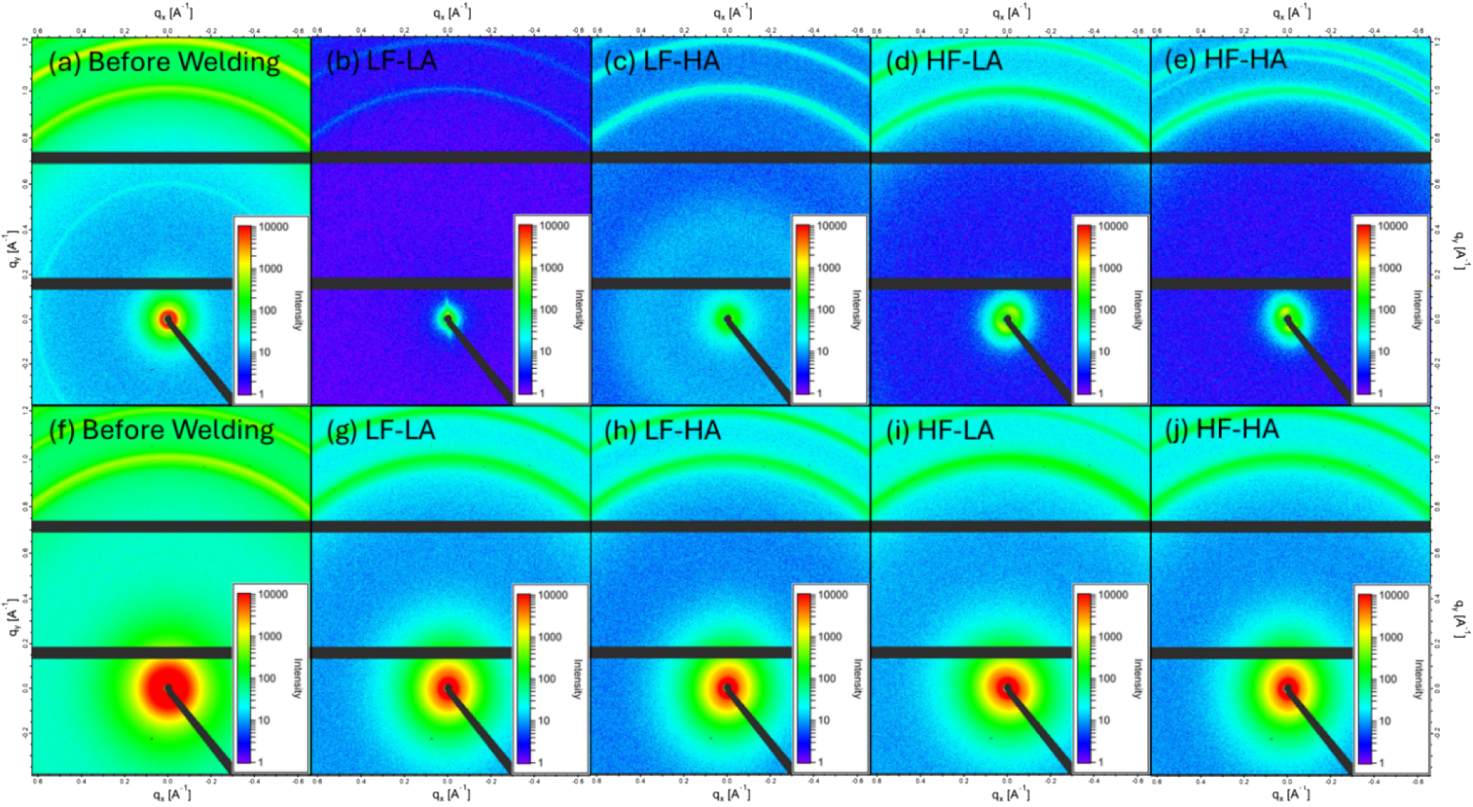
2D WAXS images of PP films (before welding and collected from the interface after welding) in the top row (a–e) and 2D images of MWCNT/PP (f–j) films (before welding and collected from the interface after welding) in the bottom row. LF-LA (500 N, 38.1 μm), LF-HA (500 N, 54 μm), HF-LA (1500 N, 38.1 μm), and HF-HA (1500 N, 54 μm).

The Lorentz-corrected SAXS intensity profiles (*Iq* ^2^ vs *q*) for pure PP and MWCNT/PP films before and after welding provide insights into the structural evolution during USW (Figure 9). For PP films before welding (Figure 9a) and MWCNT/PP films before welding (Figure 9b), the intensity profiles show a relatively low scattering signal, indicating a less developed long-range order in the crystalline domains. After welding under high force-high amplitude (HF-HA) conditions, the PP films (Figure 9c) and MWCNT/PP films (Figure 9d) exhibit a significant increase in intensity at low-*q* values, suggesting enhanced lamellar ordering and crystallinity. The shift in scattering intensity and the absence of secondary peaks indicate a transformation in the nanoscale morphology, likely driven by strain-induced crystallization and molecular realignment during welding. Lorentz-corrected SAXS intensity profiles (*Iq* ^2^ vs *q*) for other pure PP and MWCNT/PP samples are presented in Figures S6 and S7, respectively.

**Figure 9:**
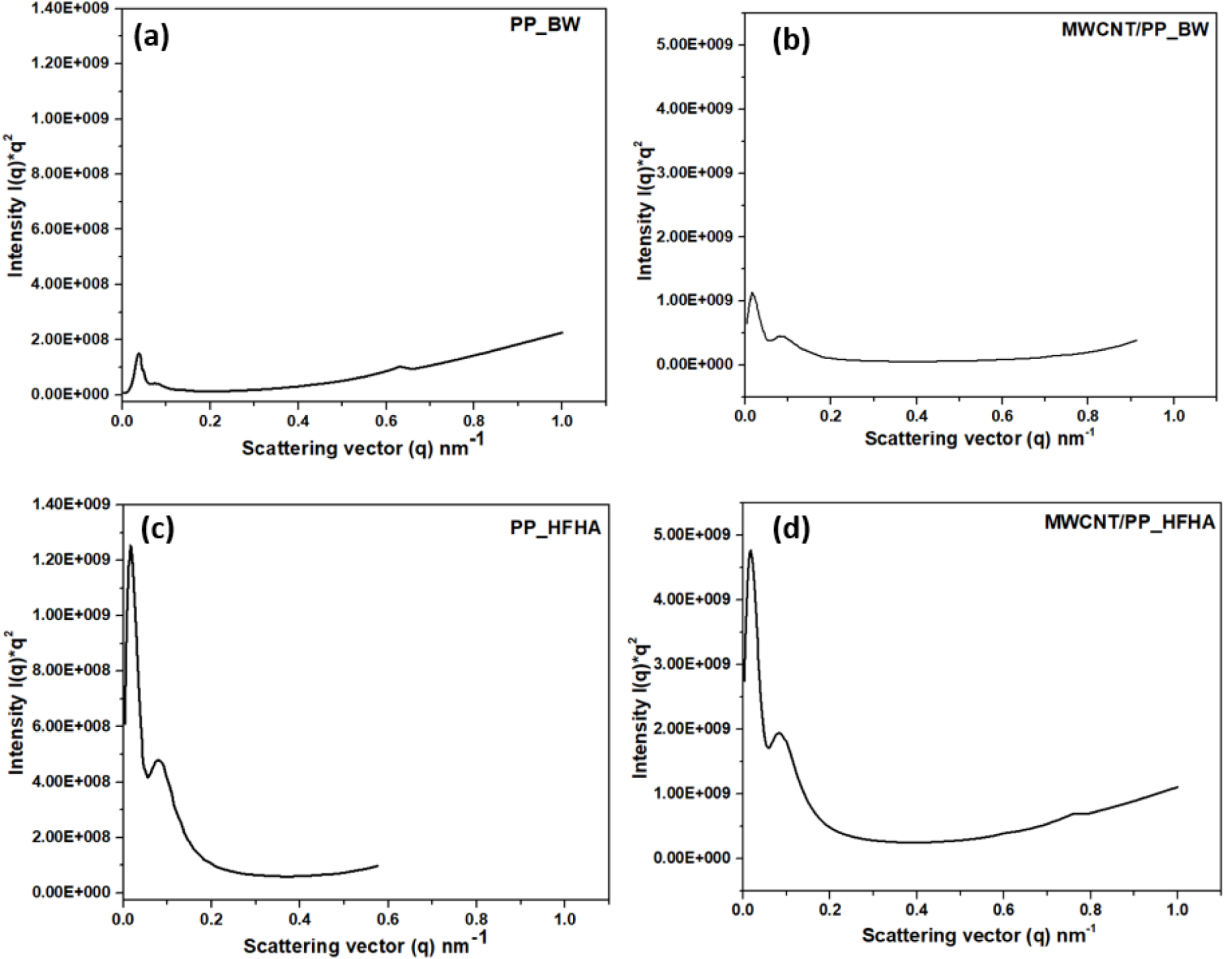
Lorentz-corrected SAXS intensity profiles (*Iq* ^2^ vs *q*) for (a) pure PP films before welding (BW), (b) 20 wt % MWCNT/PP films before welding (BW), (c) pure PP films collected from the interface after welding at high force-high amplitude (HF-HA; 1500 N, 54 μm) and (d) 20 wt % MWCNT/PP films collected from the interface after welding at high force-high amplitude (HF-HA; 1500 N, 54 μm).

The crystallinity of the samples, as determined by SAXS, is listed in Tables 4 and 5. Specifically, at HF-HA settings (1500 N, 54 μm), both pure PP and MWCNT/PP samples demonstrated maximum crystallinity (54.3% and 60.2%, respectively). Conversely, at LF-LA (500 N, 38.1 μm), crystallinity was observed to be minimal for pure PP samples (40.8%) whereas for MWCNT/PP samples, LF-LA and HF-LA conditions demonstrated the lowest crystallinity values (45.3% and 42.6%, respectively).

**Table 4:** Morphological Parameters of PP Specimens before Welding and Collected Film after Welding with Different Welding Parameters by SAXS^a^

USW Parameters	*L*_p_ (nm)	*d*_c_ (nm)	d_a_ (nm)	*d*_tr_ (nm)	*X*_C_ (%)
Before Welding	6.85	3.04	2.65	1.16	44.4
LF-LA (500 N, 38.1 μm)	6.04	2.46	2.92	0.66	40.8
LF-HA (500 N, 54 μm)	7.15	3.32	2.25	1.58	46.5
HF-LA (1500 N, 38.1 μm)	6.17	2.74	2.51	0.92	44.5
HF-HA (1500 N, 54 μm)	8.04	4.36	2.03	1.65	54.3

a *L*_p_: long period of lamellar structure, *d*_c_: thickness of crystalline layer, *d*_a_: thickness of amorphous layer, *d*_tr_: thickness of transition layer, *X*_c_: crystallinity calculated from relationship (*L*_p_/*d*_c_).

**Table 5:** Morphological Parameters of 20 wt % MWCNT/PP Specimens Before Welding and Collected Film After Welding with Different Welding Parameters by SAXS

USW Parameters	*L*_p_ (nm)	*d*_c_ (nm)	*d*_a_ (nm)	*d*_tr_ (nm)	*X*_C_ (%)
Before Welding	6.92	2.98	2.15	1.78	43.2
LF-LA (500 N, 38.1 μm)	7.04	3.18	2.75	1.10	45.3
LF-HA (500 N, 54 μm)	8.25	3.89	2.55	1.8	47.2
HF-LA (1500 N, 38.1 μm)	7.17	3.05	2.65	1.46	42.6
HF-HA (1500 N, 54 μm)	8.14	4.9	1.8	1.43	60.2

### 3.5 Effect of USW Parameters on Crystallinity and Strain-Induced Crystallinity (SIC)

The examination of films through various methods, including DSC, WAXD, and SAXS, aimed to understand the influence of process parameters on crystallinity. All techniques revealed a similar trend for crystallinity (Figures 3, 4b, 6, Tables 4 and 5), with WAXD generally indicating higher values over the other methods, particularly in the welded specimens with pure PP films. This demonstrates a correlation between process parameters and crystallinity, especially when the amplitude was increased from low to high (38.1 to 54 μm), while the welding force remained constant. Specifically, employing high welding force and vibration amplitude (HF-HA) led to the highest crystallinity at the welded interface. This suggests there could be a threshold amplitude value triggering SIC at the interface, particularly notable when high amplitude is applied. In SIC, mechanical strain can initiate the formation or rearrangement of crystals within a material. While USW predominantly relies on localized friction and heat to fuse materials, the application of mechanical force can induce crystal alignment and promote nucleation, thus affecting crystallinity. Additionally, the energy provided by mechanical forces facilitates the formation of new crystal nuclei, fostering crystallization despite rapid cooling, for the material investigated in this study. Regarding the difference in crystallinity between pure PP and MWCNT/PP films, as shown in Figure 3 for DSC measurements, a two-sample *t* test was conducted between both cases at each condition. The test yielded a *p*-value <0.05 for films before welding, LF-LA, and HF-HA, confirming that the observed difference is statistically significant and indicating that the addition of MWCNTs has a measurable effect on crystallinity enhancement.

Previous research on ultrasonic consolidation of CF/PPS layers indicated a similar trend, where an increase in welding force during USW enhanced crystallinity in the PPS matrix.^52^ It was expected that elevated welding force would promote molecular alignment and packing, consequently increasing crystallization within the PPS material. However, cooling rates were not considered in this study, which could play a role in crystallization behavior. In contrast, previous work from Koutras et al. showed that for PPS, a welding force of 300 N and a vibration amplitude of 51.8 μm produced moderate crystallinity, whereas a force of 1000 N and an amplitude of 86.2 μm resulted in predominantly amorphous PPS.^19^ In the present study, it is expected that despite the fast cooling rates measured for high conditions (HF-HA, Section 2.3 and Figure S1), the PP matrix remained above the melting temperature under high strain (high vibration) long enough to promote SIC. These findings show the effect of welding parameters on the crystallinity of PP under high cooling rates, emphasizing the need for tailored welding conditions, especially since changes in crystallinity can influence mechanical properties.^19,33,53^

To further generalize the effect of USW parameters on crystallinity, *X*_*c*_ obtained from DSC measurements was plotted with respect to the corresponding welding time and energy values (Figure 10). Time and energy were selected for comparison as they are welding control modes commonly used in the literature.^4,5^ As welding force and amplitude decreased (from HF-HA to LF-LA), welding time and energy significantly increased. It was also observed that low force conditions (LF-HA and LF-LA) led to longer welding times and higher energy values, compared to high force conditions (HF-HA and HF-LA). For the pure PP films (Figure 10a,c), a higher crystallinity was observed at shorter welding times and lower energy inputs (corresponding to HF-HA), which aligns with the earlier observations in DSC, FTIR, WAXD, and SAXS data. As welding time or energy increased (e.g., LF-HA or LF-LA), crystallinity decreased, likely caused by the extended thermal exposure facilitating chain mobility and relaxation, therefore reducing the overall crystalline content due to the dominance of amorphous phase formation. The MWCNT/PP composite films exhibited similar trends (Figure 10b,d), with higher crystallinity observed for shorter times and lower energy (HF-HA), favoring crystalline phase development despite the presence of MWCNTs. Overall, there was a significant decrease in crystallinity when welding time or energy increased, while *X*_c_ for intermediate time or energy values (HF-LA and LF-HA conditions) was within standard deviation.

**Figure 10:**
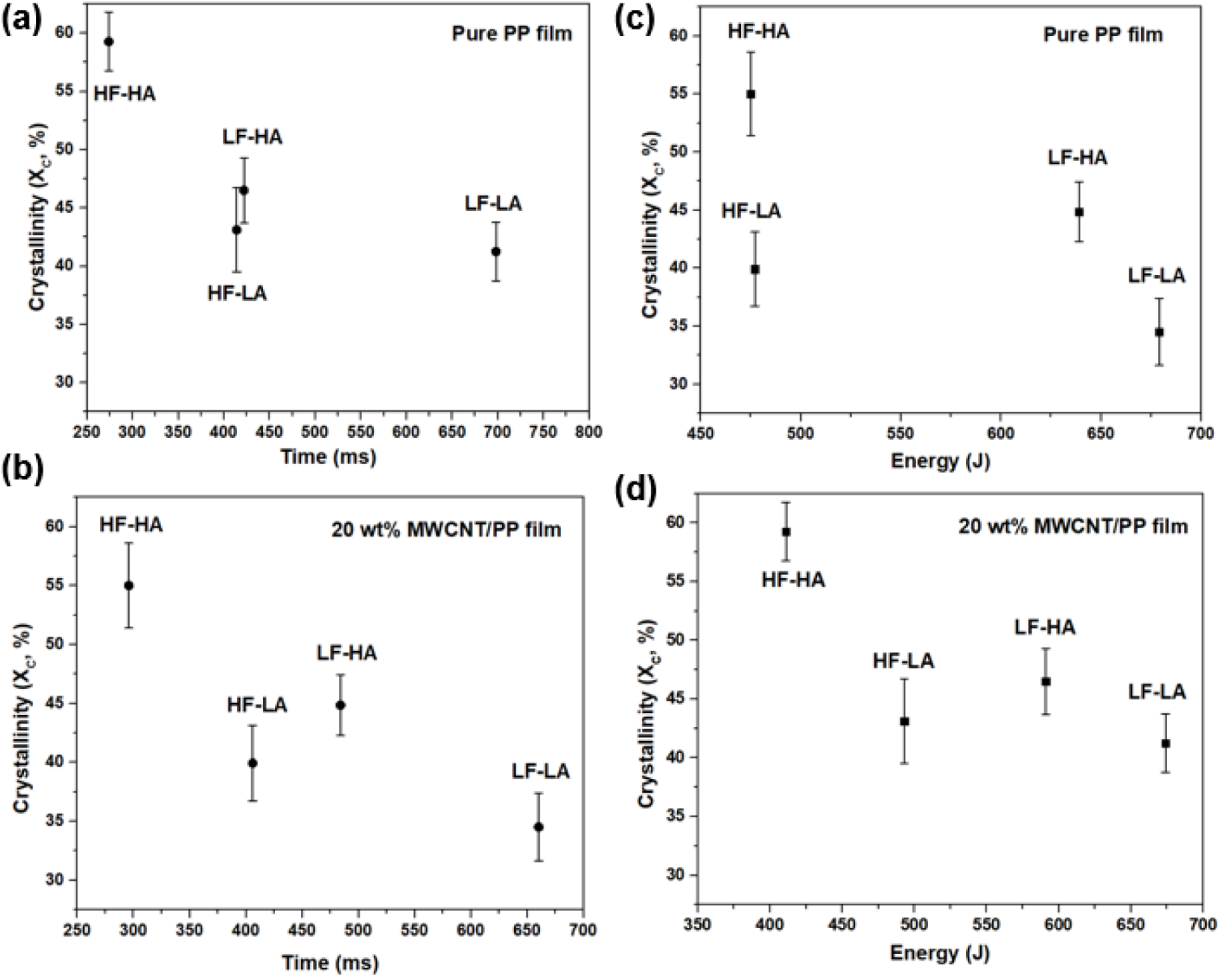
Comparison of crystallinity (X_C_, %) measured from DSC for (a) PP films with respect to welding time, (b) 20 wt % MWCNT/PP films with respect to welding time, (c) PP films with respect to welding energy, and (d) 20 wt % MWCNT/PP films with respect to welding energy, for all welding process parameters. LF-LA (500 N, 38.1 μm), LF-HA (500 N, 54 μm), HF-LA (1500 N, 38.1 μm), and HF-HA (1500 N, 54 μm).

Summarizing the findings presented in the previous sections, Figure 11 schematically illustrates the effect of USW processing conditions on crystallinity (Figure 11a) and crystallite size, molecular structure, and anisotropy (Figure 11b) at the interface. As a general recommendation for GF/PP joints, it can be expected that similar behavior would be observed when the USW process is controlled with longer time or higher energy (under the same force and amplitude parameters), but it is important to note that higher amplitudes would likely increase the average crystallinity at the welded interface due to SIC.

**Figure 11:**
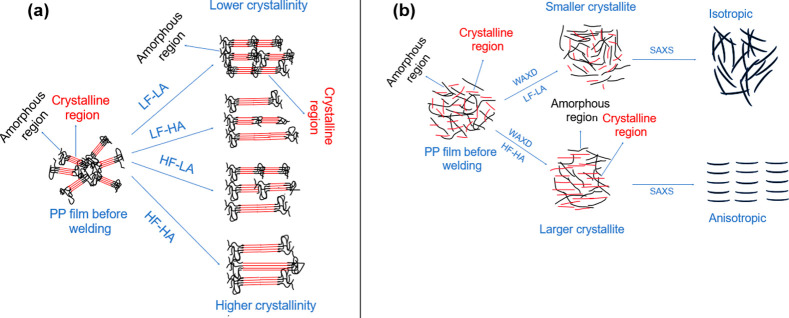
Schematic representation of the effect of ultrasonic welding parameters on PP film at the interface with respect to (a) crystallinity and (b) crystallite size, molecular structure, and anisotropy.

### 3.6 Effect of USW Parameters on Crystalline Structure and Anisotropy

Figure 11b shows a schematic representation of the main outcomes from WAXD and SAXS measurements with respect to crystalline structure (crystallite size) and anisotropy. The WAXD analysis in Tables 2 and 3 showed that lower welding force (LF) and amplitude (LA) led to a reduced crystal size along all planes (110, 040, 130, 111, 041) in both pure PP films and MWCNT/PP films, while higher force (HF) and amplitude (HA) resulted in larger crystal sizes. Conversely, no significant alteration in *d*-spacing values was detected due to variations in welding parameters, suggesting that the crystal structure of PP remained unaffected by changes in ultrasonic welding conditions. It is a fundamental property of the polymer’s crystalline phase and is determined by its intrinsic molecular structure. It is expected that USW alters the degree of crystallinity and crystallite size by influencing nucleation and growth dynamics, but it does not modify the inherent lattice parameters of PP, which are dictated by its chemical composition and molecular arrangement.

From the SAXS analysis presented in Tables 4 and 5, increasing force and amplitude during USW resulted in enhanced thickness of crystalline lamellae (*d*_c_), thickness of transition layer (*d*_tr_), and elongation of the long period (*L*_p_) within the material’s structure. This is especially significant with an increase of amplitude for the same force value. This phenomenon indicates a direct impact of mechanical strain on the crystallization process, suggesting SIC. Higher levels of mechanical deformation influence molecular reorganization, facilitating nucleation and growth of crystals. Consequently, the observed changes reflect a shift toward a more ordered molecular arrangement (more anisotropic), accompanied by a decrease in the amorphous layer thickness.^54,55^ SAXS and WAXD analyses confirmed that both pure PP and MWCNT/PP films exhibited isotropic structures before welding, characterized by uniform scattering patterns shown in Figure 7a,f (SAXS), Figure 8a,f (WAXS), and no directional dependence. However, after welding, the scattering patterns shifted distinctly toward anisotropy, particularly under HF-HA conditions, as schematically presented in Figure 11b. This transition is indicative of SIC, where mechanical deformation during welding aligns polymer chains and promotes anisotropic crystalline growth. The SAXS-derived parameters, such as lamellar long periods (*L*_p_) and crystallinity (*X*_c_), further validate this transformation. More accurately, the *L*_p_ increased significantly from 6.92 nm in isotropic MWCNT/PP films (Table 5) to 8.14 nm in anisotropic films after welding at high force and amplitude (HF-HA).

## 4 Conclusion

This work examined the effects of ultrasonic welding force and amplitude on several fundamental characteristics at the interface of ultrasonically welded GF/PP joints with both pure PP films and MWCNT/PP films: degree of crystallinity, crystalline phases, crystallite size and spacing, lamellar structure and anisotropy, and molecular changes. This investigation employed diverse analytical techniques (DSC, FTIR, WAXD, and SAXS) to explore the impact of process parameters on crystallinity. The results for all techniques consistently showed the same trend between parameters and crystallinity levels. For instance, based on DSC results, higher welding force and amplitude (HF-HA) significantly enhanced crystallinity, compared to low force-low amplitude (LF-LA), achieving 55% for welds with pure PP films and approximately 60% for MWCNT/PP films, compared to 35% and 41%, respectively, before welding. Notably, amplitude influenced the crystallinity at the welded interface more significantly compared to the force. This suggests strain-induced crystallization, where mechanical strain can initiate crystal formation or rearrangement in the matrix. While ultrasonic welding primarily relies on localized friction and heat for melting, the introduction of mechanical strain can trigger crystal alignment and nucleation, thereby influencing crystallinity despite fast cooling rates.

A generalized comparison between USW parameters using welding time and energy showed that higher crystallinity was obtained at shorter welding times and lower energy inputs (corresponding to HF-HA conditions). As welding time or energy increased (e.g., LF-HA or LF-LA), crystallinity decreased. This is likely caused by the prolonged thermal exposure facilitating chain mobility and relaxation, and consequently reducing the overall crystalline content, while the amorphous phase formation increased. This behavior was observed for pure PP films and MWCNT/PP films used as energy directors.

WAXD analyses showed that lower welding force and amplitude led to smaller crystal sizes along all planes, but higher force and amplitude (HF-HA) resulted in larger crystal sizes. No significant change in *d*-spacing values was detected for different welding parameters, suggesting that the crystal structure of PP remained unaffected by the ultrasonic welding conditions. SAXS analyses confirmed that both pure PP and MWCNT/PP films exhibited isotropic arrangement before welding, characterized by uniform scattering patterns, and no directional dependence. However, after welding, the scattering patterns shifted distinctly toward anisotropy, particularly under HF-HA conditions. This suggests strain-induced crystallization, where mechanical deformation during welding aligns polymer chains and promotes anisotropic crystalline growth. Other SAXS-derived parameters, such as lamellar long periods (*L*_p_) and crystallinity (*X*_c_), confirmed this transformation as well.

Overall, USW parameters can be selected to potentially tailor crystallinity and crystalline structure at the welded interface of thermoplastic composites. This study identified a range of welding parameters that significantly affected both crystallinity and structure, showing that amplitude had the most significant influence, for PP composites. In the future, this study will be expanded by performing in situ SAXS measurements at elevated temperatures and different cooling rates, as well as other polymer matrices and composite systems. This would support optimization of welding parameters or exploration of alternative welding techniques to assess crystallinity and mechanical performance at the interface.

## Supplementary Material



## Notes

Temperature slope during cooldown after USW process computed via backward differentiation; DSC curves for the system containing pure PP films before welding; DSC curves for the system containing pure PP films extracted from the interface after welding; DSC curves for the system containing 20 wt % MWCNT/PP films before welding and after welding; examples of Lorentz corrected SAXS scattering intensity profiles; (PDF)
