# Supplementary material for: Tailoring the Crystallinity of Ultrasonically Welded Interfaces in Glass Fiber-Reinforced Thermoplastic Composites
Source: ACS Appl Eng Mater. 2025 May 1;3(5):1455–67. doi: 10.1021/acsaenm.5c00281 (PMC12105025; doi:10.1021/acsaenm.5c00281)
Supplement: Supplementary file 1 [file em5c00281_si_001.pdf]

Supporting Information for

Tailoring the Crystallinity of Ultrasonically Welded Interfaces in Glass Fiber-Reinforced Thermoplastic Composites

Md Asmat Ullah[1], Wencai Li[1,2], Miriam Siebenbuerger[3], Felipe Savella[1], and Genevieve Palardy[1*]

[1]Department of Mechanical and Industrial Engineering, Louisiana State University

3261 Patrick F. Taylor Hall

Baton Rouge, LA 70803, United States

[2]Department of Mechanical Engineering, University of Michigan

2350 Hayward Street

Ann Arbor, MI 48109, United States

[3] Center for Advanced Microstructures and Devices, Louisiana State University

Baton Rouge, LA 70803

* Corresponding author: gpalardy@lsu.edu

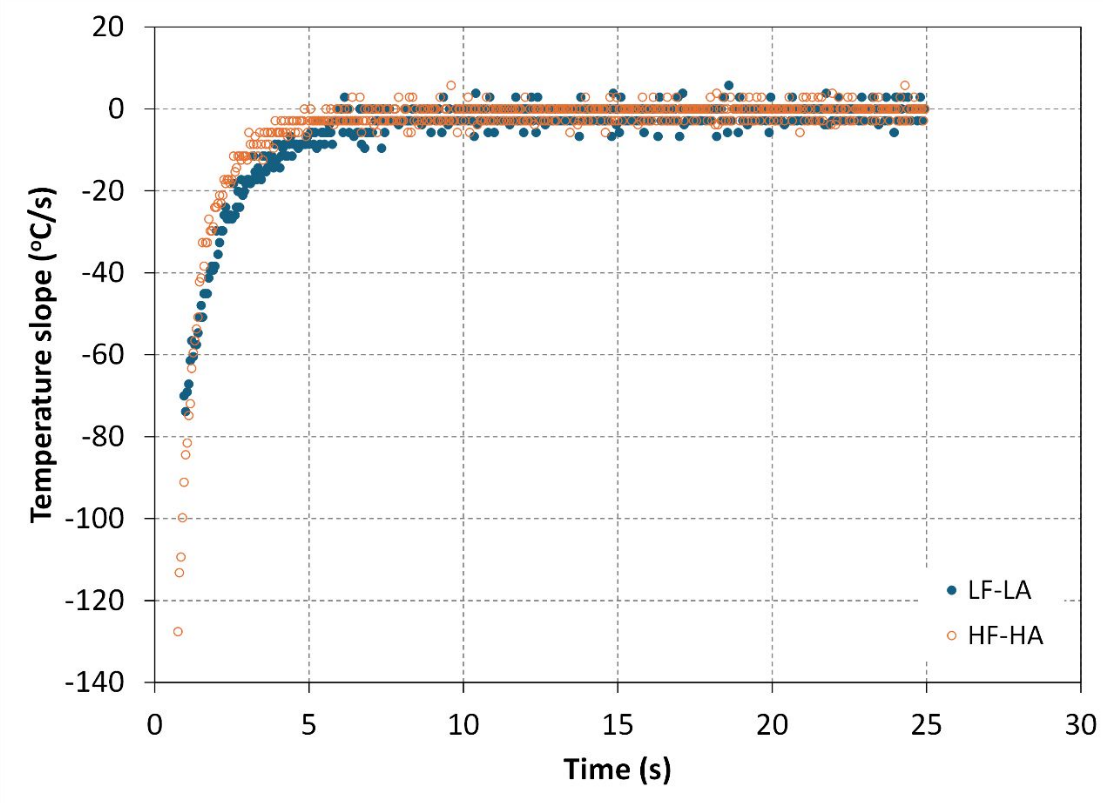

Figure S1. Temperature slope during cooldown after USW process computed via backward differentiation for low force – low amplitude (LF-LA) and high force – high amplitude (HF-HA) cases.

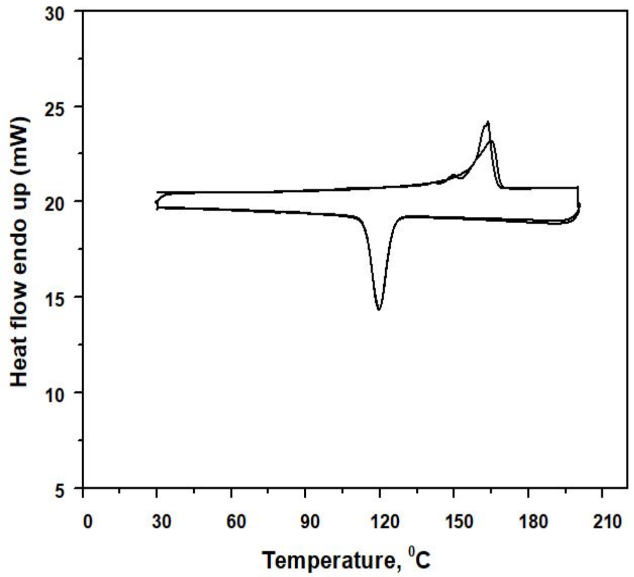

Figure S2. DSC curves for the system containing pure PP films before welding. The procedure was a ramp rate from 30 ℃ to 200 ℃ at 10 ℃ min^{-1}.

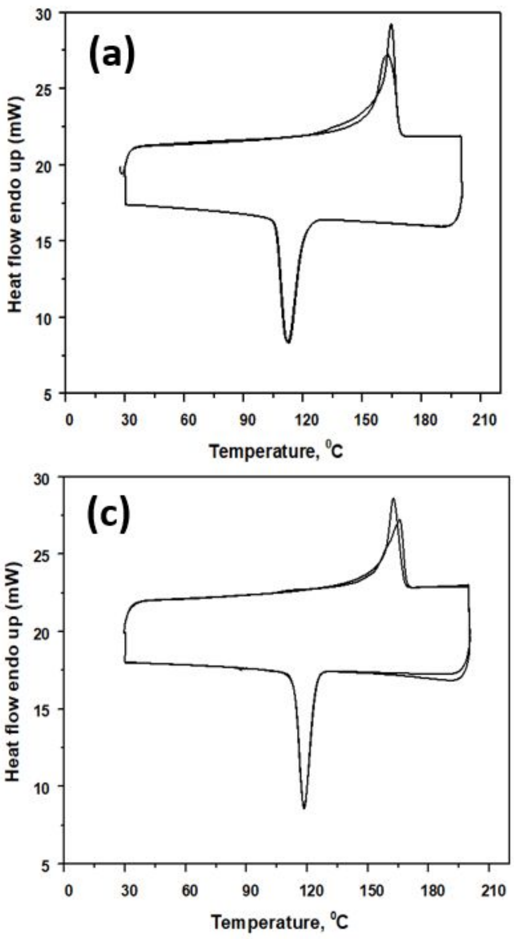

Figure S3. DSC curves for the system containing pure PP films extracted from the interface after welding at (a) low force and low amplitude (500 N, 38.1 μm), (b) low force and high amplitude (500 N, 54 μm), (c) high force and low amplitude (1500 N, 38.1 μm) and (d) high force and high amplitude (1500 N, 54 μm). The procedure was a ramp rate from 30 ℃ to 200 ℃ at 10 ℃ min^{-1}.

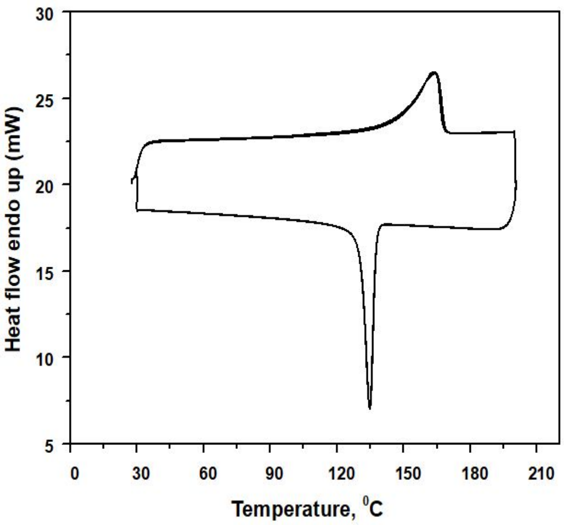

Figure S4. DSC curves for the system containing 20 wt% MWCNT/PP films before welding. The procedure was a ramp rate from 30 ℃ to 200 ℃ at 10 ℃ min^{-1}.

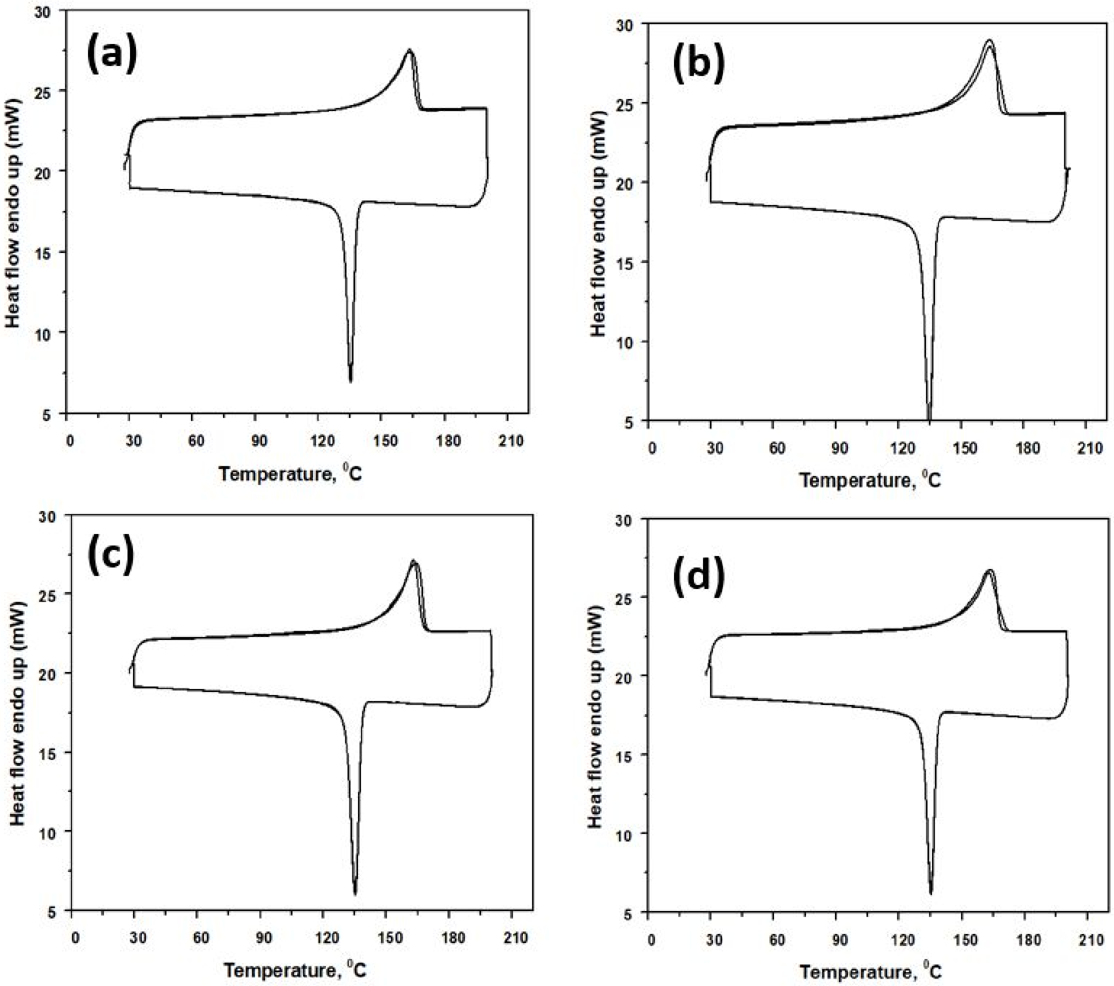

Figure S5. DSC curves for the system containing 20 wt% MWCNT/PP films extracted from the interface after welding at (a) low force and low amplitude (500 N, 38.1 μm). (b) low force and high amplitude (500 N, 54 μm), (c) high force and low amplitude (1500 N, 38.1 μm) and (d) high force and high amplitude (1500 N, 54 μm). The procedure was a ramp rate from 30 °C to 200 °C at 10 °C min^{-1}.

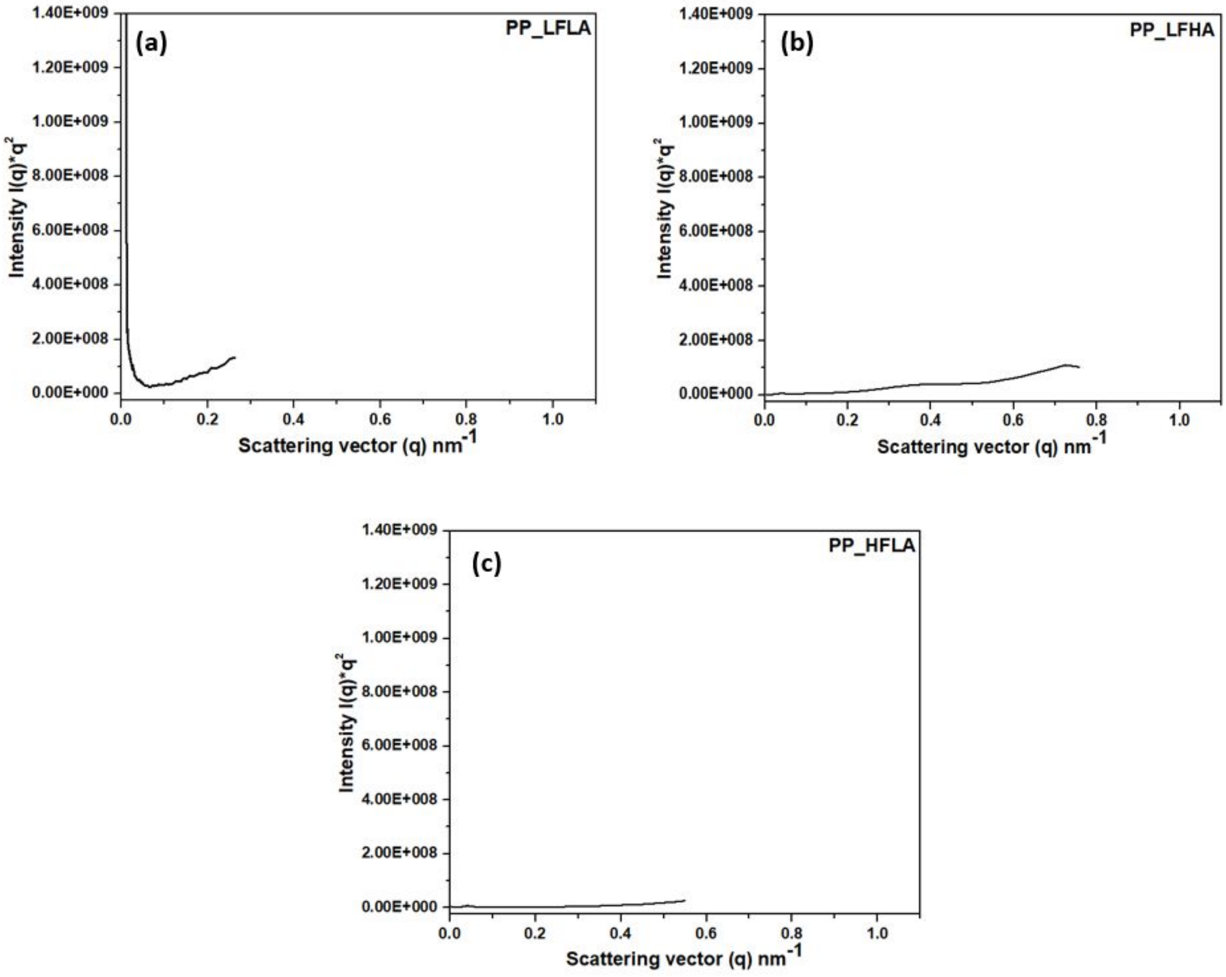

Figure S6. Examples of Lorentz corrected SAXS scattering intensity profiles for the system containing pure PP films extracted from the interface after welding at (a) low force and low amplitude (500 N, 38.1 μm), (b) low force and high amplitude (500 N, 54 μm) and (c) high force and low amplitude (1500 N, 38.1 μm).

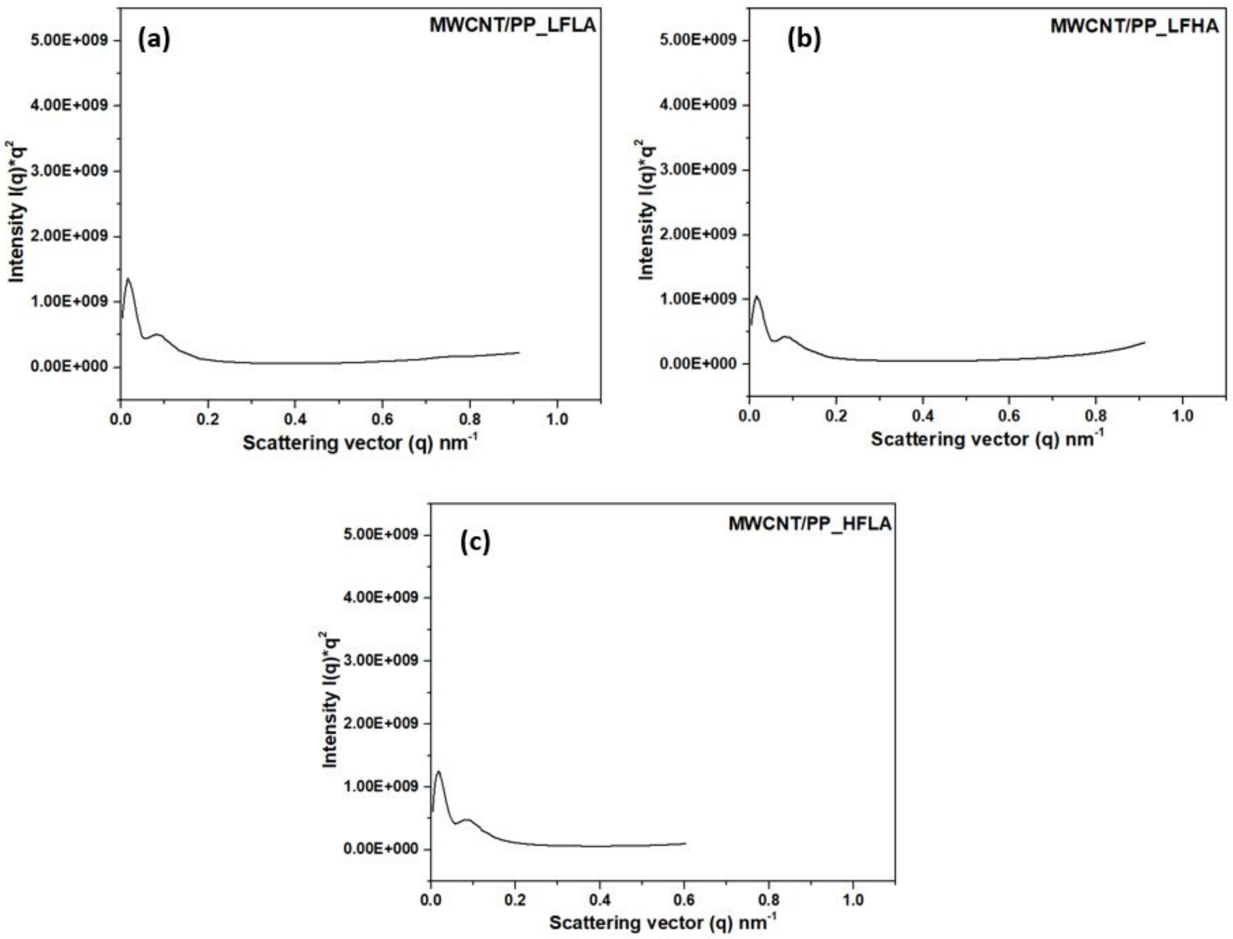

Figure S7. Examples of Lorentz corrected SAXS scattering intensity profiles for the system containing 20 wt% MWCNT/PP films extracted from the interface after welding at (a) low force and low amplitude (500 N, 38.1 µm), (b) low force and high amplitude (500 N, 54 µm) and (c) high force and low amplitude (1500 N, 38.1 µm).
